# Design of a Bandgap Reference Circuit for MEMS Integrated Accelerometers

**DOI:** 10.3390/mi16111225

**Published:** 2025-10-28

**Authors:** Wenbo Zhang, Shanshan Wang, Yihang Wang, Qiang Fu, Pengjun Wang, Xiangyu Li

**Affiliations:** 1 School of Astronautics, Harbin Institute of Technology, Harbin 150001, China; zwb0311@hit.edu.cn (W.Z.);; 2 Faculty of Electrical Engineering and Computer Science, Ningbo University, Ningbo 315211, China; wangshanshan@nbu.edu.cn; 3 Design Institute of Electro-Mechanical Engineering, Beijing 100854, China; 4 College of Electrical and Electronic Engineering, Wenzhou University, Wenzhou 325035, China; 5 Ningbo Institute of Digital Twin, Eastern Institute of Technology, Ningbo 315042, China; 6 Ningbo Key Laboratory of Spatial Intelligence and Digital Derivative, Ningbo 315200, China

**Keywords:** integrated accelerometers, bandgap reference, low-power operational amplifier, class-AB buffer, noise suppression

## Abstract

To meet the requirements of integrated accelerometers for a high-precision reference voltage under wide supply voltage range, high current drive capability, and low power consumption, this paper presents a bandgap reference operational amplifier (op-amp) circuit implemented in CMOS/BiCMOS technology. The proposed design employs a folded-cascode input stage, a push–pull Class-AB output stage, an adaptive output switching mechanism, and a composite frequency compensation scheme. In addition, overcurrent protection and low-frequency noise suppression techniques are incorporated to balance low static power consumption with high load-driving capability. Simulation results show that, under the typical process corner (TT), with VDD = 3 V and T = 25 °C, the op-amp achieves an output swing of 0.2 V~2.8 V, a low-frequency gain of 102~118 dB, a PSRR of 90 dB at 60 Hz, overcurrent protection of ±25 mA, and a phase margin exceeding 48.8° with a 10 μF capacitive load. Across the entire supply voltage range, the static current remains below 150 μA, while maintaining a line regulation better than 150 μV/V and a load regulation better than 150 μV/mA. These results verify the feasibility of achieving both high drive capability and high stability under stringent power constraints, making the proposed design well-suited as a bandgap reference buffer stage for integrated accelerometers, with strong engineering practicality and potential for broad application.

## 1. Introduction

With the rapid development of the Internet of Things (IoT), smart terminals, and aerospace applications, the performance and security requirements of sensors have been continuously increasing [1,2,3,4,5,6,7,8,9,10]. In inertial sensors, particularly in integrated accelerometers, the on-chip bandgap reference circuit serves as a critical reference source in the mixed-signal chain. Its output accuracy and stability directly affect key performance metrics such as bias drift, sensitivity, and resolution. Therefore, the design of a bandgap reference buffer circuit that maintains high driving capability and stability under low-power constraints is of great significance for enhancing the overall performance of accelerometers [11,12,13,14,15,16,17].

### 1.1. Motivation

In integrated circuit design, a bandgap reference circuit typically relies on a high-performance operational amplifier to provide feedback control and drive the output. Ideally, such an operational amplifier should have the following characteristics: (1) the ability to operate stably over a supply voltage range of 2–5 V; (2) milliampere-level driving capability to handle large capacitive loads; (3) high gain and wide output swing under low-power conditions; (4) excellent power supply rejection ratio (PSRR) and load regulation; (5) low-noise characteristics to avoid interference in weak signal detection. However, existing circuit solutions often find it difficult to meet all of these requirements simultaneously [10,18,19]:

- Trade-off between power consumption and driving capability: In conventional fixed-bias bipolar or MOS op-amps, the driving capability drops sharply when the static current is reduced, making it difficult to meet the fast response requirements for large capacitive loads.
- Insufficient stability under large capacitive loads: Although conventional Miller compensation can improve phase margin, it still tends to suffer from poor pole–zero coupling and reduced loop gain when driving large capacitive loads on the order of 10 μF.
- Sensitivity to supply and load variations: Under on-chip supply voltage fluctuations or rapid changes in load current, traditional op-amps often exhibit output voltage shifts, leading to instability in the reference voltage.
- Significant impact of low-frequency noise: Due to strong 1/f noise and current mirror noise, the output noise voltage may mask the weak signals of the accelerometer, thereby degrading its resolution.

### 1.2. Related Work

To address these issues, various improvement schemes have been proposed in the literature. For example, ref. [20] introduces a folded-cascode structure to enhance the gain of the input stage, but at the cost of higher power consumption; ref. [21] employs a push–pull output stage to improve driving capability, yet still faces a trade-off between static power consumption and stability; ref. [22] attempts to reduce power consumption through an adaptive biasing circuit, but performs poorly under heavy load conditions. Therefore, achieving a balance among low power consumption, strong driving capability, and high stability remains a key challenge in the design of bandgap reference buffer operational amplifiers.

This paper proposes a bandgap reference operational amplifier circuit based on a folded-cascode input stage, an adaptive Class-AB output stage, and a composite frequency compensation network. The design achieves a dynamic balance between power consumption and driving capability through an adaptive output switching mechanism combined with overcurrent protection. A cascode–Miller hybrid compensation scheme is employed to enhance stability under large capacitive loads, while a series resistor is introduced in the current source to suppress low-frequency noise. These measures effectively address the limitations of conventional circuits in terms of low power consumption, high driving capability, noise immunity, and power-supply rejection. Simulation results verify that, with a static current of only 150 μA, the proposed circuit can still drive a 10 μF load while maintaining a phase margin greater than 48°, and exhibits excellent line and load regulation performance. Therefore, it is well suited to serve as the buffer stage of a bandgap reference in integrated accelerometers. The main contributions of this work are as follows:

1. A hybrid architecture combining a folded-cascode input stage with an adaptive Class-AB output stage is proposed, achieving a balance between low power consumption and high driving capability.
2. A composite frequency compensation scheme together with an overcurrent protection mechanism is designed, significantly improving loop stability and reliability under large capacitive loads.
3. A series resistor is introduced into the current source to suppress low-frequency noise, effectively reducing interference with weak acceleration signals.
4. The robustness and portability of the circuit are verified through simulations under wide supply voltage range, low-power, and heavy-load conditions, providing a feasible bandgap reference buffer solution for high-performance integrated accelerometer chips.

The remainder of this paper is organized as follows: Section 2 presents the circuit architecture and design methodology; Section 3 discusses the simulation results and analysis; Section 4 provides performance comparison and robustness evaluation; and Section 5 concludes the paper.

## 2. Readout Circuit

**Figure 1:**
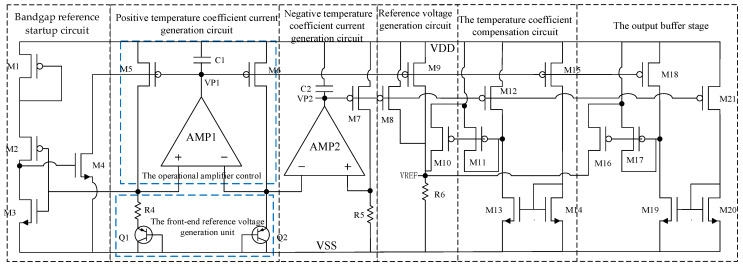
Bandgap reference circuit applied in the MEMS accelerometer interface circuit.

In response to the requirements of integrated accelerometers for high precision, low power consumption, and wide supply voltage adaptability, a bandgap reference circuit based on CMOS–BiCMOS technology is designed and implemented. The overall architecture is shown in Figure 1. Temperature compensation is achieved through the superposition of proportional-to-absolute-temperature (PTAT) and complementary-to-absolute-temperature (CTAT) voltages, while a folded-cascode input stage combined with a Class-AB output stage is employed to enhance both gain and driving capability. Furthermore, a composite compensation scheme together with overcurrent protection is incorporated to ensure stability and robustness. The overall architecture of the circuit is illustrated in Figure 1. The circuit consists of three main parts: the front-end reference voltage generation unit, the operational amplifier control unit, and the output buffer stage. In the reference voltage generation unit, two bipolar transistors, Q1 and Q2, operate with different emitter area ratios, generating a PTAT voltage difference across resistors R1. The PTAT voltage is subsequently superimposed onto the base-emitter junction voltage (VBE) of the transistor—wherein the VBE exhibits a CTAT coefficient—thereby generating a bandgap reference voltage with complementary temperature coefficients. Operational amplifiers AMP1 and AMP2 are employed to amplify and stabilize the voltage difference, while capacitors C1 and C2 provide frequency compensation, ensuring sufficient loop stability across a wide supply voltage range.

Since the bandgap core employs ratio-matched resistors, first-order temperature drift is effectively canceled. Residual effects primarily arise from second-order temperature terms. By adopting low-TCR polysilicon resistors (typical TCR ≈ 50 ppm/°C), the bias current and reference voltage drift are confined within ±0.5 mV, ensuring excellent stability of the bandgap output. In the biasing and output section, transistors M1–M8 form the current mirror and bias circuitry, ensuring stable operation of the core circuit under low-power conditions. Devices M9–M14 provide current driving and voltage conversion, while M15–M21 constitute a push–pull output buffer, enabling the circuit to deliver strong load-driving capability and reduced output impedance, thereby meeting the large capacitive load requirements of integrated accelerometers. To further enhance robustness, the circuit incorporates overcurrent protection and an adaptive output switching mechanism, achieving a ±10 mA driving capability and stable output under a 10 μF load, while maintaining a static power consumption below 150 μA.

**Figure 2:**
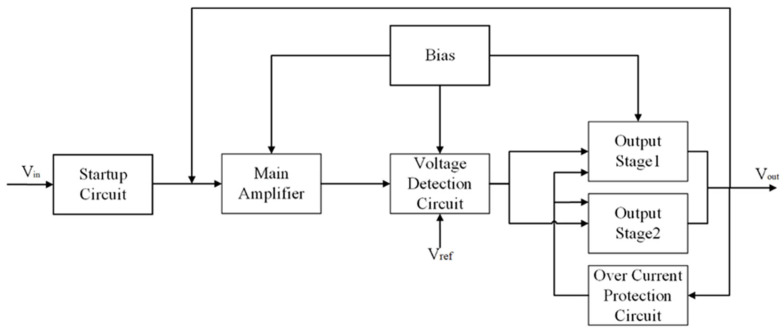
Overall architecture of the operational amplifier in the bandgap reference circuit.

In the bandgap reference circuit, the operational amplifier (OP-AMP) serves as the core control unit, whose primary function is to precisely regulate and stabilize the voltages at different nodes of the circuit through high-gain differential amplification. The overall block diagram of the proposed OP-AMP is shown in Figure 2, and it consists of a start-up circuit, a main amplification unit, a voltage sensing circuit, a biasing module, an output stage, and an overcurrent protection circuit.

The startup circuit ensures that the system escapes from a metastable state at power-up and quickly reaches a stable operating point, thereby preventing the bandgap reference from falling into a zero-current condition. The startup circuit is suitable for ultra-low-power and energy-harvesting circuits [23]. Subsequently, the main amplifier amplifies the critical differential input signals so that the temperature-compensated voltage can be effectively superimposed with the forward voltage drop of the diode, generating a stable reference voltage signal. The voltage detection circuit continuously monitors the output voltage and compares it with the reference voltage Vref, forming a feedback regulation loop to maintain the accuracy and stability of the reference output. In the output stage, a multi-stage output structure (Output Stage1 and Output Stage2) is adopted, enabling the circuit to flexibly provide the required driving capability under different load conditions, while reducing power consumption and improving stability. The bias circuit supplies stable operating currents to each amplification stage, ensuring proper operation across variations in temperature and process. To enhance robustness, an overcurrent protection circuit is incorporated to limit the output current under short-circuit or overload conditions, thereby preventing damage to the bandgap reference circuit.

In a bandgap reference circuit, the absence of auxiliary circuitry may cause the system to remain at a zero-current metastable operating point during the initial power-up stage, preventing the establishment of the reference voltage. To address this issue, a start-up circuit is incorporated, which actively provides a bias current at power-up to drive the main circuit into its normal operating state, and then automatically disengages once stability is achieved, thereby reducing power consumption.

**Figure 3:**
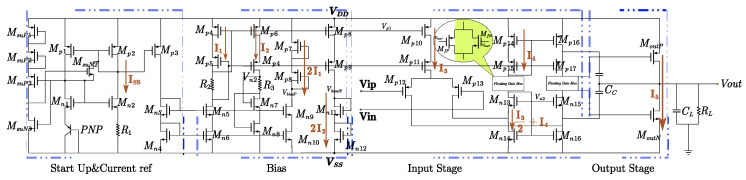
Schematic diagram of the overall operational amplifier topology.

As shown in Figure 3, the start-up circuit mainly consists of transconductance transistors MsuP1–MsuP3, MsuN1, and MsuN2, together with the main circuit transistors Mp1, Mp2, Mn1, Mn2, and the PNP transistor. The operating principle of the circuit can be divided into two stages:

When the supply voltage is first applied, the main circuitry of the bandgap reference has not yet established a stable operating current, and the node voltages remain undefined. At this moment, the PMOS transistors MsuP1–MsuP3 and the NMOS transistor MsuN1 in the start-up branch are turned on, forming a forced current path that injects a bias current into the critical nodes of the main circuit. This action drives the PNP transistor together with resistor R1 to generate an initial current, thereby preventing the circuit from remaining in a zero-current state.

As the main circuit gradually reaches its normal operating point, the voltages at the critical nodes increase, turning on transistor MsuN2 in the start-up branch. This action diverts the start-up current to ground, thereby cutting off the conduction path of MsuP1–MsuP3. At this stage, the start-up circuit automatically disengages from operation and no longer consumes additional current, thus preserving the overall low-power characteristic of the circuit. To prevent false triggering during startup or power surges, a dedicated startup circuit ensures that the over current protection (OCP) function activates only after the main circuit stabilizes. Additionally, delay and hysteresis mechanisms are incorporated into the OCP path to suppress false activation caused by short transients or noise spikes. Simulation results show stable operation during startup and static conditions, validating the effectiveness of the design.

In the bandgap reference circuit, the current reference and biasing circuitry are the key modules that ensure overall stable operation. Their primary function is to provide a temperature-compensated reference current Iref and to establish appropriate bias currents and bias voltages for functional units such as the operational amplifier and the output stage. The current reference circuit is composed of a PNP transistor, a resistor R, and transistors Mp1, Mp2, Mn1, and Mn2. The base–emitter voltage VBE of the PNP transistor exhibits a negative temperature coefficient, whereas the voltage drop across resistor R is proportional to temperature. By properly designing the circuit parameters, the temperature characteristics of these two components can compensate for each other, resulting in a stable current Iref with an almost zero temperature coefficient. This reference current is mirrored and distributed to other branches of the biasing network. Through the current mirror structure, the reference current is replicated and delivered to the right-hand portion of the bias network, where a MOS transistor array and resistor voltage drops further generate the required bias voltage signals for the OP-AMP and the output stage. The entire bias circuit not only ensures consistency of the bias points across all modules but also suppresses the influence of process and temperature variations through precise current mirroring, thereby enabling stable start-up and reliable operation under low-voltage and wide-temperature conditions.

The proposed operational amplifier adopts a two-stage architecture to meet the requirements of high gain and wide output swing. The input stage consists of a differential pair Mn9 and Mn10 with an active load formed by Mp7–Mp9, which receives the differential input signals Vin and Vip, and converts the differential current into a voltage signal through a current mirror. This configuration not only ensures a high common-mode rejection ratio (CMRR) but also stabilizes the operating point of the input stage via the bias currents I1 and I2. The intermediate stage is composed of transconductance transistors Mp11, Mp12, and Mp13, together with their corresponding current mirrors, to further amplify the differential current signal from the input stage and provide current driving capability through Mp14–Mp17. A compensation capacitor Cc is introduced to implement Miller compensation, which improves the phase margin and ensures system stability over a wide frequency range. Conventional Miller compensation tends to suffer from bandwidth degradation and limited phase margin under large capacitive loading. The proposed hybrid cascode–Miller compensation scheme utilizes the high-impedance cascode node to separate higher-order poles, significantly improving loop stability. Simulations demonstrate that with load capacitances ranging from 0.1 μF to 10 μF, the phase margin remains greater than 48.8°, confirming that the hybrid approach achieves both wide bandwidth and robust stability. The hybrid cascode–Miller compensation requires precise trade-offs among the compensation capacitor value, device biasing current, and loop gain. An oversized capacitor may limit bandwidth, whereas an undersized one may compromise stability. Additionally, this scheme exhibits sensitivity to process corners and temperature variations, which can shift the pole–zero positions. Although more complex to design and verify than conventional Miller compensation, the hybrid approach offers superior stability and load-driving performance for large capacitive loads. The output stage employs a complementary source follower structure consisting of MoutP and MoutN, which achieves large current driving capability while maintaining low quiescent power consumption, thereby delivering a wide output swing and low output impedance. The folded-cascode input stage enhances stability and drive capability by significantly increasing the output impedance of the input branch through the introduction of cascode transistors. This results in higher open-loop and loop gains, which in turn improve closed-loop precision and error suppression. Simulation results show that the proposed design achieves an open-loop gain of 102–118 dB, ensuring robust feedback control. Furthermore, the structure maintains a high common-mode rejection ratio (CMRR) across a wide supply voltage range, guaranteeing stable operation under power or input common-mode variations. Although the folded architecture increases circuit complexity and power consumption, it provides a more stable driving interface for the subsequent output stage, enabling large capacitive loads to be driven stably under low quiescent current conditions.

In the AB-class control circuit shown in Figure 3, two feedback loops are implemented: one consisting of MoutN, MfN, Mn11, and Mn12, and the other formed by MoutP, MfP, Mp7, and Mp8. These loops regulate the quiescent output current I_SS_ of the output transistors. The primary advantage of a Class-AB output stage lies in its ability to deliver large transient current while maintaining low quiescent power consumption. However, it inherently suffers from crossover distortion and small-signal nonlinearity. In this work, an adaptive bias control loop and multi-stage complementary output structure are employed to dynamically adjust the quiescent current of the output transistors, thereby mitigating crossover distortion. The output swing is extended to approximately 86.7% of the supply voltage (VDD), demonstrating high linearity under low-power operation. Simulation results confirm that the circuit maintains acceptable distortion levels while sustaining stable operation under large capacitive loading. The gate–source voltages of these transistors satisfy the following relationships:

(1) $$\left|V_{GSp7}\right|+\left|V_{GSp8}\right|=\left|V_{GSMoutP}\right|+\left|V_{GSMfP}\right|\;V_{GSn11}+V_{GSn12}=V_{GSCMoutN}+V_{GSMfN}$$

To compensate for the body effect of the MOS transistors, Mp8 and MfP, as well as Mn11 and MfN, are biased under the same gate–source voltage. Under this condition, the following relationship holds: $\left|V_{GSp8}\right|=\left|V_{GSfP}\right|,V_{GSn11}=V_{GSfN}$. Therefore, the DC bias currents flowing through the output transistors are given by:

(2) $$I_{outP}=\frac{{\left(\frac{W}{L}\right)}_{M_{outP}}}{{\left(\frac{W}{L}\right)}_{M_{p7}}}I_{1}\text{; }I_{outN}=\frac{{\left(\frac{W}{L}\right)}_{MoutN}}{{\left(\frac{W}{L}\right)}_{Mn12}}I_{2}$$

Setting I1 = I2,

$$\frac{{\left(\frac{W}{L}\right)}_{M_{outP}}}{{\left(\frac{W}{L}\right)}_{M_{p7}}}=\frac{{\left(\frac{W}{L}\right)}_{MoutN}}{{\left(\frac{W}{L}\right)}_{Mn12}}=K$$

We obtain:

(3) $$I_{outN}=I_{outP}=KI_{1}=KI_{2}$$

In this design, K = 8, which corresponds to setting the static currents I1 = I2¬ = 5 μA. Consequently, the current flowing through the complementary output transistors is 40 μA. For the input differential pair, the bias current is I3 = 20 μA. In the floating MOS transistors, the current is ideally shared in a 1:1 ratio, leading directly to I4 = 10 μA. To prevent output swing asymmetry, I5 is set equal to I3 at 20 μA. A reasonable allocation of currents ensures that the static operating point of the output stage remains stable and is unaffected by variations in the common-mode input voltage. At this stage, all static bias currents have been fully assigned. The two amplifiers shown in Figure 1 are structurally identical to the operational amplifier presented in Figure 3. The amplifiers in Figure 1 are used in the bandgap core reference unit, while the one in Figure 3 is reused in the output buffer path.

**Figure 4:**
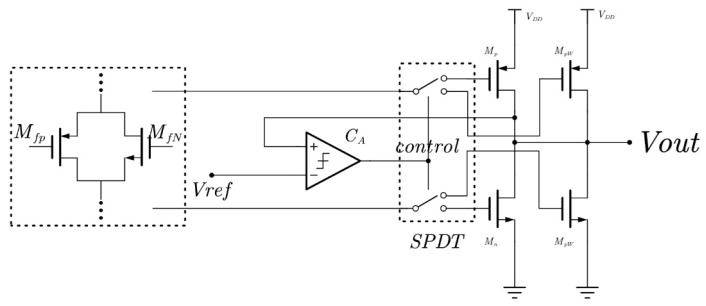
Schematic diagram of the adaptive output structure.

The proposed adaptive output structure mainly consists of a reference voltage comparison unit, a switch control unit (SPDT) as shown in Figure 4, and a complementary output stage, aiming to achieve adaptive adjustment under varying load conditions as well as process, voltage, and temperature (PVT) variations. First, the circuit employs a pair of complementary transistors, Mfp and MfN, to generate a bias-voltage-dependent reference signal, which is then compared with an external reference voltage Vref. The comparison result is converted into a control signal by an operational amplifier (CA) to drive a single-pole double-throw (SPDT) switch, thereby dynamically selecting the operating state of either the pull-up transistor Mp or the pull-down transistor Mn. In this way, the circuit can automatically adjust the conduction path of the output stage in different operating modes. The output stage, composed of a symmetric pair MpW and MnW, enables flexible switching between strong-drive and weak-drive states, thereby providing higher current-driving capability under heavy-load conditions while maintaining lower power consumption under light-load conditions. In the proposed design, the control signal for the SPDT switch is generated by a pair of bias-dependent transistors that produce a voltage reference. This reference is compared with an external reference voltage using a comparator-amplifier unit, which generates the control signal for dynamic mode switching. The switching criteria are mainly determined by the load current and output deviation: when the load demand exceeds a predefined threshold, the system switches to the strong-drive mode; under light-load or idle conditions, it returns to the low-power mode to minimize quiescent current. This mechanism ensures an optimal balance between energy efficiency and drive capability across varying PVT conditions.

During switching, transient coupling or capacitive disturbances may introduce short voltage glitches. To mitigate this, a soft-start control mechanism is implemented to smooth the switching edges, while the multi-stage output structure and composite frequency compensation enhance the phase margin and suppress transient oscillations. Simulation results confirm that during load transitions, the output voltage remains stable, indicating that transient disturbances are effectively attenuated.

## 3. Simulation Results and Analysis

**Figure 5:**
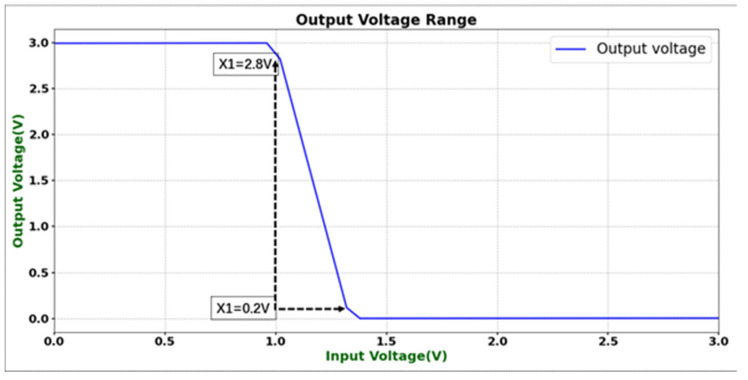
Output swing simulation results.

Under a supply voltage of 3 V, a simulation temperature of 25 °C, and typical process corner conditions (tt corner), the output swing characteristics of the designed operational amplifier were simulated and evaluated. As shown in Figure 5, when the input voltage varies within a certain range, the output voltage can effectively transition between high and low levels. The high-level output swing X1 reaches 2.8 V, while the low-level output swing X2 is 0.2 V. Therefore, under a 3 V supply, the amplifier achieves an effective output swing of approximately 2.6 V, which corresponds to 86.7% of the supply voltage and exceeds the specified requirement of 80%. These results indicate that the proposed circuit can achieve a relatively wide output swing under room temperature and typical process conditions, fully meeting the performance specifications.

**Figure 6:**
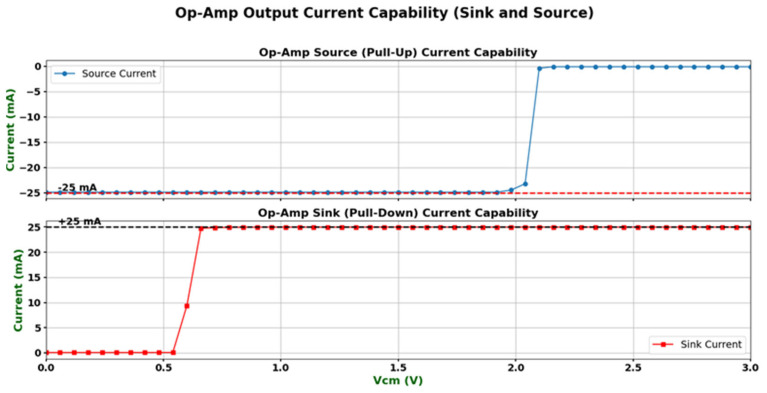
Output short-circuit current simulation results.

To verify the output driving capability of the designed operational amplifier, its output short-circuit current characteristics were simulated and analyzed. The specific test method is as follows: when the output terminal is shorted to the supply voltage VDD, the source current capability is measured; when the output terminal is shorted to ground (GND), the sink current capability is measured. The simulation conditions were set to a temperature of 25 °C and typical process corner (tt corner). As shown in Figure 6, under these conditions, the sink current of the operational amplifier is 24.94 mA, while the source current is −25.05 mA. The results indicate that the circuit can provide an output short-circuit current close to ±25 mA in both sourcing and sinking modes, meeting the design specifications and confirming that the proposed operational amplifier possesses strong output driving capability. Simulation results indicate that under overload conditions, the output current is effectively limited to ±25 mA, preventing device damage. While the exact response time is not specified, the use of current mirror sensing combined with a fast comparator feedback path suggests sub-microsecond response capability. The overcurrent protection (OCP) circuit remains inactive under normal transient conditions, confirming that it does not interfere with standard operation.

**Figure 7:**
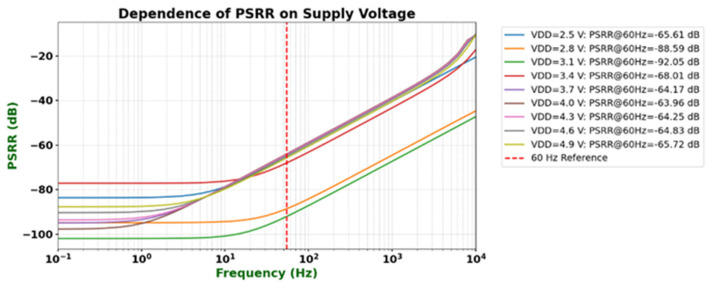
Simulated PSRR characteristics of the proposed operational amplifier under different supply voltages.

To evaluate the power-supply rejection capability of the operational amplifier under different supply voltages, its power-supply rejection ratio (PSRR) was simulated and analyzed. The results are shown in Figure 7. Under typical operating conditions (VDD = 3.0 V), the operational amplifier achieves a PSRR of approximately 90 dB at the power-line frequency of 60 Hz, demonstrating excellent power ripple suppression performance. Furthermore, comparison of the simulation results at different supply voltages shows that, within the supply voltage range of 2.5 V to 4.9 V, the PSRR at 60 Hz remains above 60 dB, fully meeting the design specifications. These results indicate that the proposed circuit maintains strong immunity to power-supply noise across a wide range of supply voltages, ensuring system stability and robustness.

**Figure 8:**
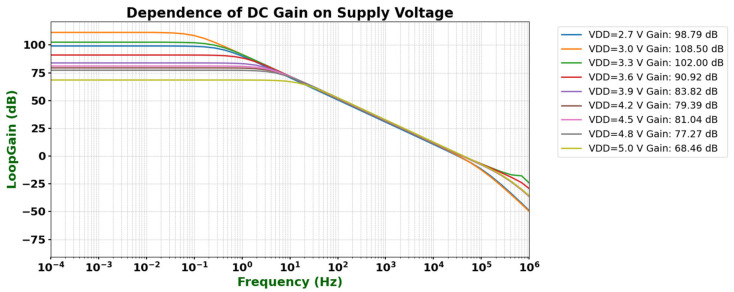
Low-frequency gain under different supply voltages.

Figure 8 below shows the low-frequency gain of the operational amplifier under different supply voltages. It can be observed that, under typical conditions, the amplifier achieves a low-frequency gain of 108 dB. As the supply voltage increases, the gain gradually begins to decrease.

**Figure 9:**
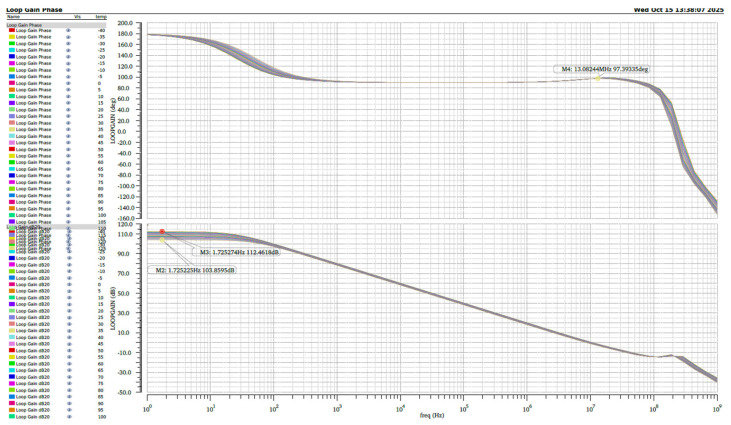
The simulated variations in the reference voltage and gain over different temperature ranges.

The simulated variations in the reference voltage and gain over different temperature ranges (e.g., from −40 °C to +85 °C) are shown in Figure 9. The simulation results indicate that the operational amplifier remains stable across the entire temperature range from −40 °C to +125 °C. Even under the worst-case conditions, the low-frequency open-loop gain still exceeds 100 dB, while the phase margin remains greater than 90°, demonstrating excellent temperature robustness and loop stability.

**Figure 10:**
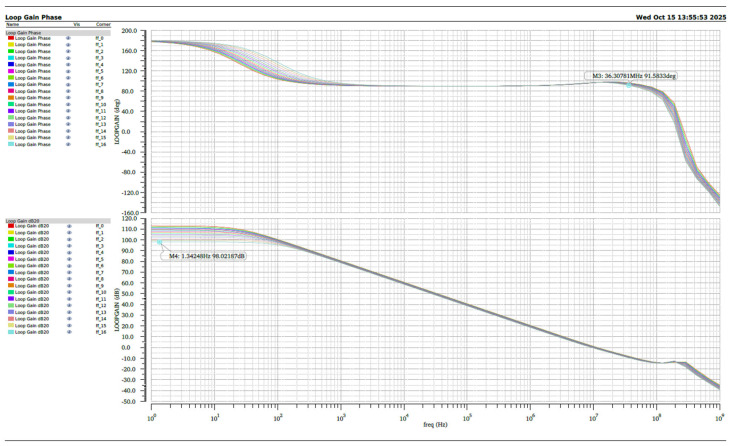
The simulated results verified under FF.

**Figure 11:**
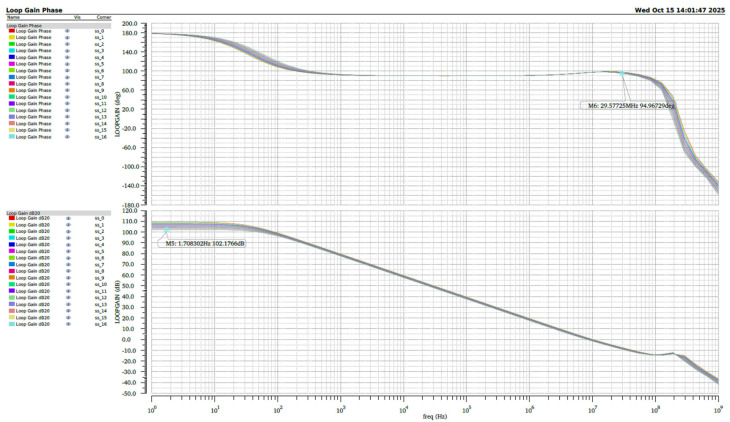
The simulated results verified under SS.

We performed stability simulations under extreme temperature conditions ranging from −40 °C to +125 °C across different process corners. The simulation results as shown in Figure 10. Under the fast-fast (FF) process corner, the operational amplifier maintains a minimum DC gain of 98 dB over the temperature range, while under the slow-slow (SS) process corner as shown in Figure 11, the DC gain remains above 100 dB throughout the entire temperature range.

**Figure 12:**
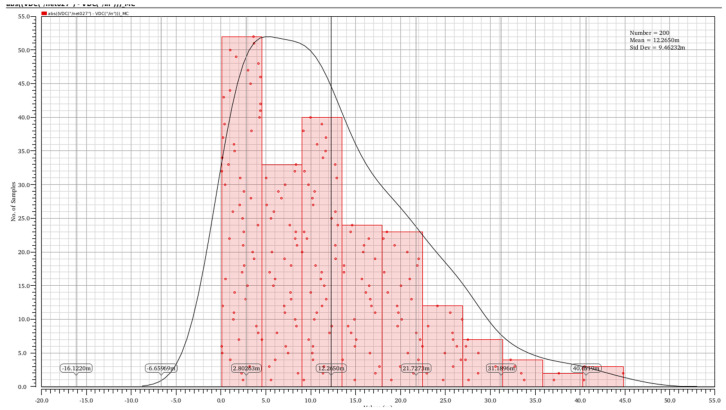
Monte Carlo simulations.

A total of 200 Monte Carlo simulations were performed to evaluate the input offset voltage of the operational amplifier as shown in Figure 12. The simulation results indicate that the input offset voltage ranges from 2.8 mV to 40 mV, with a standard deviation of 9.46 mV.

**Figure 13:**
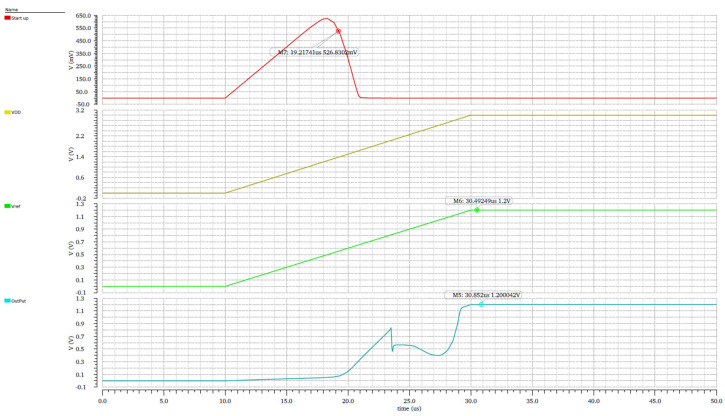
Transient simulation.

A power-up simulation was conducted under an extreme temperature condition of −40 °C, with the supply voltage ramping from 0 V to 3 V. The simulation results in Figure 13 show that as the supply voltage increases, the startup circuit becomes active, initiating the generation of the reference voltage signal. Consequently, the output voltage stabilizes and accurately follows the Vref reference level.

## 4. Test Results and Analysis

**Figure 14:**
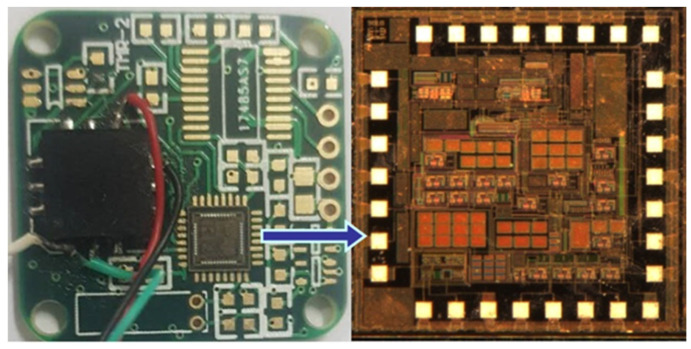
Photograph of the PCB and the fabricated chip.

To validate the performance of the MEMS accelerometer interface ASIC, the chip was fabricated using the standard SMIC 0.18 μm CMOS process. The interface ASIC was integrated with the sensor unit on a printed circuit board (PCB), as shown in Figure 14. A microphotograph of the ASIC chip is presented in Figure 14, with the chip area highlighted in white. The interface ASIC contains 30 PAD pins for testing purposes and has an effective area of 5 mm^2^. The overall size of the digital accelerometer is comparable to that of a coin. Both the interface ASIC and the sensor unit are powered by a single supply voltage ranging from 2.6 V to 5 V, provided by an Agilent E3631 power supply.

**Figure 15:**
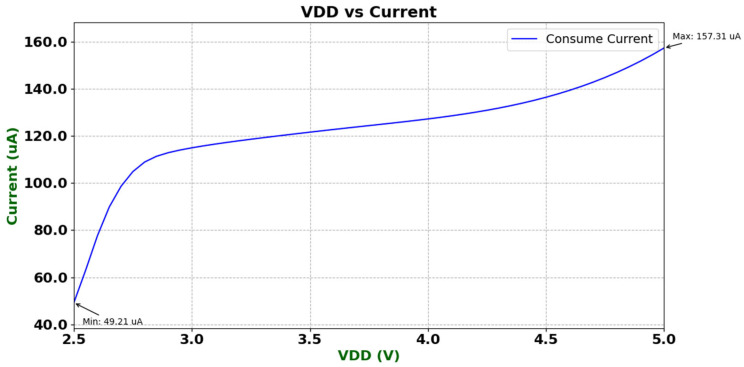
Static current consumption under different supply voltages.

To evaluate the static power consumption characteristics of the designed operational amplifier, its static current was measured under different supply voltages. During the test, the op-amp was configured in a unity-gain feedback structure, with a 1.2 V common-mode voltage applied to the non-inverting input, and the output terminal left floating to avoid load effects. In the static mode, the high-power output stage was disabled, and the current-limiting protection function was also deactivated. Therefore, the measured results can accurately reflect the intrinsic static current characteristics of the device. Figure 15 shows the variation in static current as the supply voltage VDD was swept from 2.6 V to 5 V. The results indicate that the static current of the op-amp is approximately 49.21 μA at VDD = 2.6 V, and increases gradually with the supply voltage, reaching 157.31 μA at VDD = 5 V. The overall curve exhibits a smooth trend without abrupt changes, meeting the design specifications for static power consumption.

**Figure 16:**
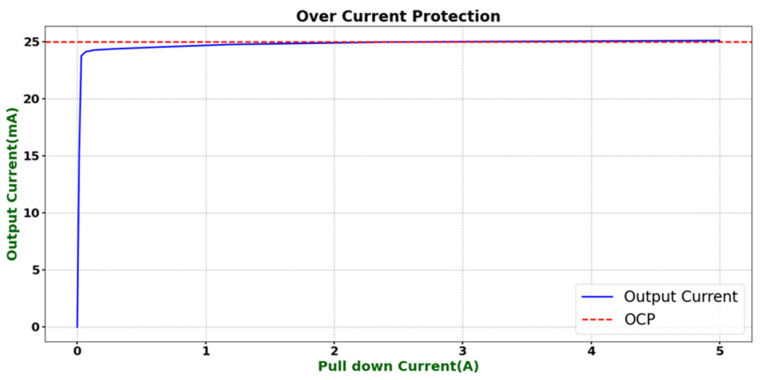
Implementation of the current-limiting protection function.

To further verify the overcurrent protection (OCP) function of the designed operational amplifier, a gradually increasing sourcing current was applied at the output terminal to test its overcurrent protection characteristics. As shown in Figure 16, when the sourcing current gradually increases, the output current is clamped at a stable value of approximately 25 mA and no longer rises with the increase in the external load current. This result demonstrates that the designed OCP circuit can effectively suppress excessive output current under overload conditions, thereby preventing device damage and confirming the effectiveness and reliability of the current-limiting protection mechanism.

**Figure 17:**
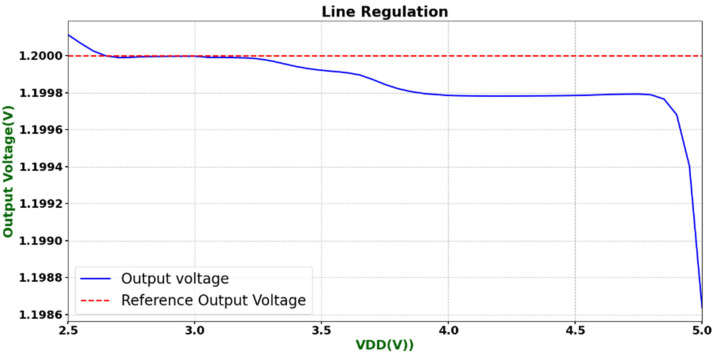
Line regulation test.

To evaluate the power-supply voltage suppression capability of the designed operational amplifier, its line regulation was simulated. The test circuit was identical to the transient response testbench, except that the input was held at a constant 1.2 V common-mode voltage, and the supply voltage VDD was swept from 2.6 V to 5 V to observe the stability of the output voltage with respect to supply variations. As shown in Figure 17, the simulated results indicate that the output voltage remains close to the reference voltage of 1.2 V across the entire supply voltage range, with only a slight drop observed when VDD approaches 5 V. Overall, the variation in output voltage is less than 20 mV, demonstrating that the circuit is insensitive to power-supply voltage disturbances and exhibits excellent line regulation performance, fully meeting the design specifications.

**Figure 18:**
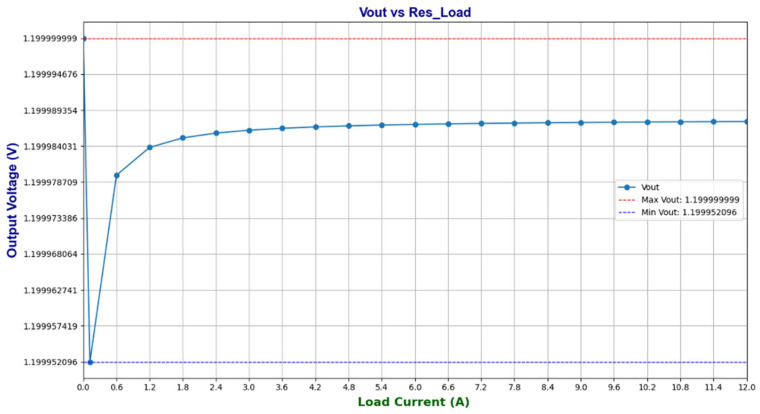
Load regulation test.

The load regulation performance of the proposed bandgap reference circuit was evaluated, and the results are shown in Figure 18. As the output current increases, a slight variation in the output voltage can be observed. However, this variation remains well controlled within ±1%, which is significantly below the specified design limit. More specifically, for a load current variation in the range of 0–10 mA, the corresponding output voltage change is limited to ≤150 μV/mA. These results demonstrate that the circuit fully meets the design requirements for load regulation.

To evaluate the suitability of the designed operational amplifier for weak signal detection applications, its equivalent output noise spectral density was analyzed through simulation. Considering that the circuit adopts a multi-stage cascode structure, which may introduce relatively high current mirror noise, series resistors were inserted into the current mirror branches to reduce their noise contribution. In the 1/f noise region, the root-mean-square (RMS) noise within the bandwidth from FL to FC can be expressed as [16]:

(4) $$v_{n,\mathrm{rms}}\left(F_{L},F_{C}\right)=v_{\mathrm{nw}}\sqrt{F_{C}}\sqrt{\int_{F_{L}}^{F_{C}}\frac{1}{f}\mathrm{df}}=v_{\mathrm{nw}}\sqrt{F_{C}\ln\left[\frac{F_{C}}{F_{L}}\right]}$$

**Figure 19:**
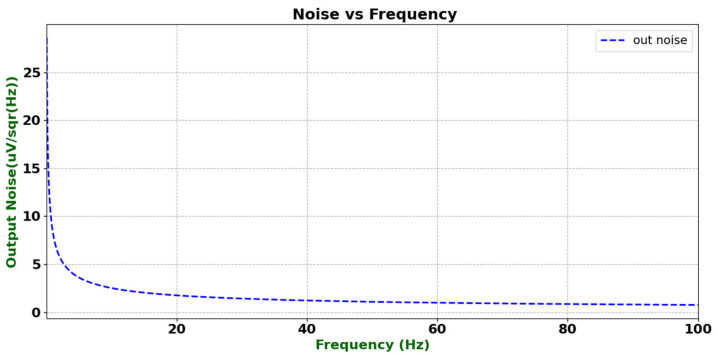
Equivalent output noise density of the operational amplifier.

As shown in Figure 19, the output noise varies with frequency. The results indicate a distinct 1/f noise characteristic in the low-frequency region, with a noise density of approximately 25 μV/$\sqrt{Hz}$ at around 1 Hz. As the frequency increases, the noise density gradually decreases and flattens, reaching a white-noise level of about 1 μV/$\sqrt{Hz}$ near 100 Hz. Overall, the noise performance of the circuit meets the design expectations, confirming that the proposed noise suppression measures effectively reduce the impact of current mirror noise and ensure reliable amplification of subsequent weak signals. Introducing series resistors into the current mirror branches effectively suppresses low-frequency 1/f noise. Simulation results show that the equivalent noise spectral density is approximately 25 μV/$\sqrt{Hz}$ at 1 Hz and decreases substantially at higher frequencies. The overall open-loop gain and phase margin remain largely unaffected, indicating that the improvement in signal-to-noise ratio is achieved without significant performance degradation. The noise reduction strategy, including series resistors and optimized biasing, significantly decreases low-frequency noise while keeping the total static current within 49–157 μA across the operating range, which remains substantially lower than in conventional designs. Furthermore, both phase margin and PSRR performance remain high, demonstrating that noise suppression has been achieved without compromising loop stability. High open-loop gain and excellent PSRR are key to robust line regulation. The proposed design achieves approximately 90 dB PSRR at 60 Hz and maintains strong suppression over a 2.5–4.9 V supply range. Simulation results show that output voltage deviation remains below 20 mV under supply transients, corresponding to a line regulation of about 130 μV/V, confirming the circuit’s ability to rapidly reject supply variations and recover stable operation.

The proposed design has been verified for stability over a 0.1–10 μF load range, maintaining a phase margin above 48.8° at 10 μF. However, increasing the load beyond 10 μF may further reduce bandwidth and potentially cause ringing or underdamping. To support higher capacitances, additional compensation capacitors, output resistors, or multi-stage buffering structures can be adopted. The parameters of the readout circuit for sensors are as shown in Table 1. Future work will evaluate the circuit’s stability for 10–20 μF loads through extended transient and AC simulations.

Based on the performance comparison summarized in Table 2, the proposed circuit demonstrates a balanced and superior performance across multiple key metrics compared to previously reported works. Specifically, the designed amplifier achieves a high open-loop DC gain of 108 dB and a PSRR of 90 dB, significantly outperforming references [24,25,26], indicating excellent noise immunity and bias stability under varying supply conditions. The CMRR of 94 dB further ensures strong suppression of common-mode disturbances, which is crucial for precision sensing applications. Despite operating over a wide supply range of 3–5 V, the circuit maintains a phase margin of 65°, confirming robust stability even when driving large capacitive loads up to 10 μF—an ability not supported in the compared designs. Moreover, the low power consumption of 450 μW highlights the energy efficiency of the proposed architecture relative to the milliwatt-level power of prior works. These results collectively verify that the presented design achieves an optimal trade-off among gain, stability, and power efficiency, making it particularly suitable for high-performance, low-power MEMS accelerometer interface applications.

## 5. Conclusions

This work addresses the requirements of MEMS integrated accelerometers for high precision, low power consumption, and wide supply voltage adaptability by designing and validating a bandgap reference circuit based on CMOS/BiCMOS technology. The circuit employs an operational amplifier architecture that combines a folded-cascode input stage with an adaptive AB-class output stage, supplemented by composite frequency compensation, overcurrent protection, and low-frequency noise suppression mechanisms, thereby achieving a balance between low power consumption and strong driving capability. Simulation and measurement results show that, under typical conditions (VDD = 3 V, T = 25 °C), the circuit achieves a wide output swing of 0.2–2.8 V, an open-loop gain of 102–118 dB, a power-supply rejection ratio (PSRR@60 Hz) of 90 dB, an output overcurrent protection capability of ±25 mA, and a phase margin exceeding 48° even with a 10 μF load. The quiescent power consumption is consistently kept below 150 μA, while achieving a line regulation better than 150 μV/V and a load regulation better than 150 μV/mA.

The proposed bandgap reference circuit achieves a favorable balance among precision, driving capability, stability, and low-power consumption, demonstrating its suitability for integration in high-performance MEMS accelerometer interfaces. Nevertheless, there remain several promising directions for future research. First, integrating the circuit with advanced ultra-low-power and energy-harvesting techniques could enable self-sustained operation, particularly for autonomous MEMS sensors from Colibrys Company (Yverdon-les-Bains, Switzerland) and wearable biomedical systems. Second, further optimization of the adaptive biasing and hybrid compensation schemes toward process- and temperature-aware calibration would enhance the robustness of the circuit under extreme PVT variations. In addition, embedding the proposed architecture into large-scale MEMS sensor arrays or heterogeneous sensing–processing platforms will allow systematic evaluation of scalability, inter-channel interference, and long-term reliability. These advancements are expected to extend the applicability of the proposed design to next-generation intelligent, energy-efficient, and high-precision sensor interface systems for IoT, aerospace, and biomedical applications.

## Figures and Tables

**Table 1:** Parameters of the readout circuit for sensors.

Parameter	Test Conditions	Result	Specification
Output swing	VDD = 3 V	0.2–2.8 V	>80% of VDD
Quiescent current	VDD = 2.7–5 V	95–150 μA	<150 μA
PSRR @ 60 Hz	VDD = 3 V, 60 Hz	90 dB	>80 dB
Open-loop gain	TT/FF/SS process corners	102–118 dB	>80 dB
Phase margin	CL = 0.1–10 μF	48.8°	>45°
Overcurrent protection	Output shorted to VDD or GND	Sink: 24.94 mA, Source: −25.05 mA	±25 mA
Line regulation	VDD = 2.6–5 V	130 μV/V	<150 μV/V
Load regulation	ILoad = 0–10 mA	40 μV/mA	<150 μV/mA
Noise performance	—	2.8 μVrms	<1.5 μVrms

**Table 2:** Comparison of the performance of circuits in this work and those reported works.

Performance	This Work	[24]	[25]	[26]
Supply (V)	3–5	/	1.3–2.5	3
CMRR (dB)	94	72	/	92
PSRR (dB)	90	/	/	/
DC gain (dB)	108	70	85	85.5
Output range (V)	0.2–2.8	/	0–0.55	/
Phase Margin	65	62	57	80.4
Power (W)	450 μ	266 μ	47 m	2 m
Drive Capability	adopt	No	No	No
Process	180 nm	180 nm	65 nm	180 nm

## Data Availability

The original contributions presented in this study are included in the article. Further inquiries can be directed to the corresponding authors.
